# Effect of Dentin Biomodification Using Naturally Derived Collagen Cross-Linkers: One-Year Bond Strength Study

**DOI:** 10.1155/2013/918010

**Published:** 2013-08-27

**Authors:** Carina S. Castellan, Ana K. Bedran-Russo, Alberto Antunes, Patricia N. R. Pereira

**Affiliations:** ^1^Department of Dental Materials, University of Sao Paulo, Avenida Professor Lineu Prestes 2227 Cidade Universitaria, 05508-000 Sao Paulo, SP, Brazil; ^2^Department of Restorative Dentistry, College of Dentistry, University of Illinois at Chicago, 801 S Paulina Street, Chicago, IL 60612, USA; ^3^Department of Operative Dentistry, University of Brasilia, Campus Universitário Darcy Ribeiro—70910-900 Brasília, DF, Brazil

## Abstract

*Purpose.* This study investigated the long-term resin-dentin bond strength of dentin biomodified by proanthocyanidin-rich (PA) agents. *Materials and Methods.* Forty molars had their coronal dentin exposed, etched, and treated for 10 minutes with 6.5% grape seed extract (GSE), 6.5% cocoa seed extract ethanol-water (CSE-ET), 6.5% cocoa seed extract acetone-water (CSE-AC), and distilled water (CO). Samples were restored either with One-Step Plus (OS) or Adper Single-Bond Plus (SB). Bond strength test was performed immediately or after 3, 6, and 12 months. *Results.* Higher **μ**TBS were observed for GSE immediately (SB- 62.9 MPa; OS- 51.9 MPa) when compared to CSE-ET (SB- 56.95 MPa; OS- 60.28 MPa), CSE-AC (SB- 49.97 MPa; OS- 54.44 MPa), and CO (SB- 52.0 MPa; OS- 44.0 MPa) (*P* < 0.05). CSE outcomes were adhesive system and solvent dependant. After 12 months storage SB results showed no difference among treatment types (GSE- 57.15 MPa; CSE/ET- 54.04 MPa; CSE/AC- 48.22 MPa; CO- 51.68 MPa; *P* = 0.347),while OS results where treatment dependent (GSE- 42.62 MPa; CSE/ET- 44.06 MPa; CSE/AC- 41.30 MPa; CO- 36.85 MPa; *P* = 0.036). *Conclusions.* GSE and CSE-ET agents provided enhanced immediate adhesion and stabilization to demineralized dentin after long-term storage, depending on adhesive system.

## 1. Introduction

The presence of cross-links in solubilized collagen provides strength, reinforcement, and stabilization to the fibrils [1] and is important feature of the use of collagen as biomaterial [2]. Collagen cross-links can be induced by either physical or chemical reagents; however, biocompatibility and low cytotoxicity are essential properties [3]. A more resistant, stable, and insoluble network is an important feature for a specific biofunction of collagen to serve as a stable substrate for dental adhesive restorations [4]. Type I collagen is the main constituent of the dentin matrix, and collagen fibrils of the mantle and intertubular dentin occupy most of the space filled by extracellular dentin matrix [5]. The three-dimensional network of demineralized collagen following acid etching serves as substrate for the application of primers or adhesive resins that infiltrate the interfibrillar spaces and promote mechanical retention, via hybrid layer (HL) formation after resin polymerization [6].

The stability of the adhesive interface relies on a compact and homogenous HL [7]. The incomplete infiltration and encapsulation of collagen fibrils by resin monomers [8], the elution of unpolymerized resin monomers overtime, and factors inherent to the oral environment [9] can cause exposure and disorganization of collagen fibrils and consequent degradation of the HL [10]. The use of collagen cross-linking agents is proposed to enhance mechanical properties of dentin matrix, reduce biodegradation rates of collagen, increase the properties of the dentin-resin bonds, and extend the life of adhesive restorations [4]. 

Proanthocyanidins (PA) are natural biocompatible collagen cross-linker broadly distributed in the plant kingdom and one of the most abundant secondary metabolites found in plants [11]. A high structural diversity based on four monomers molecules (catechin, ent-catechin, epicatechin, ent-epicatechin), different types of interflavonoids bonds, and the various lengths of chains, known as degree of polymerization (DPm), are unique characteristics of the agent [11]. Among all health benefits related to PA, particular attention has been given to their ability to bind to proline rich proteins [12], such as collagen, and facilitate the enzyme proline hydroxylase activity, essential for collagen biosynthesis [13].

Recent studies evaluated the ability of PA rich extracts to increase short- and long-term mechanical properties of demineralized dentin [14] and the short-term dentin-resin bond strength [15]. Grape and cocoa seed extracts are well known as rich PA sources, where PA can be readily extracted with regular and nontoxic solvents, like water, acetone, and ethanol [16]. Therefore, the purpose of this study was to investigate the effect of PAs' interaction with demineralized dentin on the long-term dentin-resin bond strengths. The null hypothesis tested was that long-term bond strength is not affected by dentin treatment, regardless of the PA source and adhesive system. Two total etch adhesive systems (One Step Plus and Adapter Single Bond Plus), two PA based extracts (grape and cocoa seed), and four different storage periods (immediately, 3, 6, and 12 months) were investigated.

## 2. Materials and Methods

The use of forty (40) sound extracted molars (*n* = 5) is commonly found in the literature [17] and it was approved by UIC IRB office (number 2009-0198). The teeth were ground flat using number 180, 320, and 600 grit silicon carbide papers (Buehler, Lake Bluff, IL) under running water to expose middle coronal dentin and create a standard smear layer.

## 3. Experimental Solutions 

Cocoa seed extract (*Theobroma cacao*) was obtained from Barry Callebaut (Lebbeke-Wieze, Belgium) and dissolved in water/solvent solutions. Grape seed extract (*Vitis vinifera*) was obtained from Polyphenolics (Madera, CA, USA) and dissolved in water. All chemicals (ethyl alcohol anhydrous, ≥99.5%, and acetone, ≥99.9%, Sigma-Aldrich, St. Louis, MO) were used without further purification. The concentration (6.5% weight by volume) and type of solvent systems, including the two independent ones for cocoa extract (50/50% ethanol-water and 30/70% acetone-water), were selected according to previous studies [14]. The two extracts were dissolved in their respective solvent system and the pH of slightly acidic solutions was adjusted to 7.2 using NaOH at room temperature. After pH adjustment, the solutions were filtered (paper filter n°6, Whatman, London, UK). The experimental solutions conditions were followed according to previous studies [18]. Neat solvent (distilled water) was used as negative controls. The teeth were divided according to the experimental solutions: GSE (distilled water), CSE-ET (ethanol-water), and CSE-AC (acetone-water) and control group (CO). The level of cross-linking was not controlled, merely analyzed regarding extract/solvent ratios. Five teeth were used per each experimental condition (*n* = 5). Information of the restorative materials and extracts are described in Table 1.

## 4. Bonding Procedures

Dentin surfaces were etched with the respective systems' etchants for 15 s, rinsed, treated for 10 min with individual solutions, and then thoroughly rinsed. Two total-etch adhesive systems with different solvents were tested: Adper Single Bond Plus, which is ethanol-water based (SB, 3M ESPE) and One Step Plus, which is acetone based system (OS, BISCO) (Table 1). Dentin surfaces were immersed on each experimental or control solution, for designed time, at room temperature without any external manipulation. The bonding procedures were carried out following manufacturers' instructions. The control group followed the same protocol using distilled water. Layers of Filtek Supreme (3M ESPE) resin composite were placed incrementally to build up a crown of approximately 4 mm high. Increments were light-cured for 40 seconds with a light-curing unit (Optilux 501; Kerr Corp.) that delivered 650 mW/cm^2^. The specimens were stored in water at 37°C for 24 hours.

## 5. Microtensile Bond Test (*μ*TBS) and Storage

After storage, the specimens were sectioned perpendicular to the adhesive-tooth interface into 0.8 × 0.8 mm beams using a slow-speed diamond wafering blade (Buehler Series 15LC Diamond; Buehler Ltd.) under constant water coolant. Approximately 20~25 beams were produced from each tooth. Five beams from the same tooth were tested at each storage time: 24 h, 3, 6, and 12 months. Specimens were stored in artificial saliva containing 1.5 mM calcium and 0.9 mM phosphate in a buffer solution of 0.1 M Tris buffer at pH 7.0 [19]. The storage media were changed every two weeks.

 After storage time elapsed, the specimens were fixed with cyanoacrylate glue (Zapit, Dental Ventures of America Inc., Corona, CA) to a jig, which was mounted on a microtensile tester (Bisco) and subjected to tensile forces at a crosshead speed of 1 mm/min until deboning. *μ*TBS (MPa) was calculated by dividing the peak force (N) by the cross-sectional area of the failed interface (mm^2^), measured by a digital caliper. Statistical analysis was performed for each adhesive system using two-way ANOVA (treatment versus storage time) followed by one-way ANOVA when needed and post hoc Tukey HSD tests. Statistical significance was set at *α* = 0.05%. 

## 6. Results

The bond strength values and one-way ANOVA noninteraction *P* values are depicted in Table 2 (SB). Data analysis revealed that the GSE showed the highest *μ*TBS values (SB- 62.9 MPa; OS- 51.9 MPa), which differed statistically (*P* < 0.005 for both) from those obtained with control group (SB- 52.0 MPa; OS- 44.0 MPa), despite of the adhesive system.

Two-way ANOVA for SB showed no statistical difference for storage (*P* = 0.250) or interaction (*P* = 0.947), but treatment influenced significantly dentin *μ*TBS (*P* < 0.000) (Table 3). After 24 hours, GSE showed the highest results, and values remained high until 6 months. There were no decreases in *μ*TBS values after 12 months stored in artificial saliva when compared to 24 hours evaluation (*P* = 0.185). When experimental groups were compared after 1 year storage, no statistical difference among them was verified (*P* = 0.347).

When OS was used, all groups showed a significant decrease in *μ*TBS results after 1 year when compared to the 24 hours evaluation (*P* < 0.000, for all groups). There was no statistical significant interaction between factors (*P* = 0.563); however, a different dentin treatment and storage time significantly affected dentin bond strength (*P* < 0.000, for both) (Table 3). GSE treatment resulted in the highest *μ*TBS results after 24 hours, 6 and 12 months evaluation, which were not statistically different than CSE/ET. After 1-year storage CSE/ET showed the highest bond strength values. 

**Figure 1:**
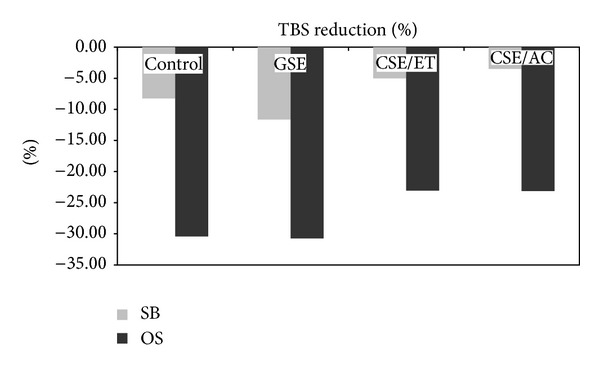
Graph illustration for the percentage of *μ*TBS reduction (%) for different dentin treatment after 12 months: GSE-grape seed, CSE/ET-cocoa seed in ethanol-water, CSE/AC- cocoa seed in acetone-water and control when used both adhesives: Single Bond Plus (SB) and One Step Plus (OS).

OS system resulted in significant decrease in long-term *μ*TBS when compared to restorations using SB. Figure 1 shows “Percentage of *μ*TBS reduction” for all experimental groups that was calculated as a percentage of the bond strength decrease using the values of bond strength immediately and after 12 months. Two-way ANOVA (adhesive system versus treatment) indicated a statistical significant difference between adhesive systems (*P* = 0.042), showing greater *μ*TBS reduction for OS when compared to SB. Interactions were not statistically different (*P* = 0.921 and *P* = 0.995, resp.).

## 7. Discussion

Clinical studies show two degradation patterns within the HL: disorganization of collagen fibrils and loss of resin from interfibrillar spaces [10, 20, 21]. The total depth of dentin demineralization is not fully infiltrated by monomers, which leaves an exposed collagen network, susceptible to hydrolytic degradation over time [9]. The removal of dentin matrix, the so-called deproteinization, has already been proposed as a possible treatment to increase bond strength longevity and decrease technique sensitivity [7, 22]. However, a long-term study revealed decrease in the deproteinized dentin-resin bonds [22]. In addition, the HL is believed to be important as a stress-buffering layer under mechanical loading [7]. Thus a stronger, stable, and resistant collagen layer is more suited substrate for current dental adhesive restorations. This study analyzed a natural, inexpensive, and promising method to enhance exogenous type I collagen cross-linking and, therefore, improve dentin-resin bonding.

Our results demonstrate that dentin biomodification did not compromise the bond strength longevity and also that effectiveness and stability of cross-linking treatment depends mostly on the source/type of PA rich extract and the adhesive system employed; thus, the null hypothesis has to be partially rejected. Whereas GSE presented higher immediate *μ*TBS values, CSE treatment outcomes were adhesive system and solvent dependant. Our immediate GSE results are in agreement with previous studies that used 1 hour dentin treatment using OS and SB adhesive systems [17, 23]. The present study observed the effect of cross-linking agents after 10 min treatment, which is much more clinically feasible, so a less time-consuming treatment is possible and most likely achievable with increased agent concentration [24]. Although a 10-minute treatment is still unsuitable for clinic reality, this study is intended to verify the laboratorial possibility to improve dentin-resin bond strength using alternative methodologies. 

Different interactions between GSE or CSE and dentin were reported in previous studies, showing superior result for GSE when it was used to improve demineralized dentin mechanical properties [14], enzymatic resistance [15], and stability for over 12 months [18]. The induction of exogenous cross-link in dentin matrix also leads to a decrease in the swelling ratio. The low swelling ratio for the treated groups may indicate that not only masking of the cleavage sites or decrease in the collagenase activity may affect the degradation of the treated dentin matrix. Low swelling ratio may indicate a decrease in the collagenase absorption and therefore assist on the matrix resistance against enzymatic degradation [15]. Cocoa seed has been described as a potential dentin bio-modification agent with a longer application time and this could be related to differences in structural composition [16]. High phase liquid chromatography (HPLC) studies show that cocoa seed has greater amount of monomers and oligomers up to pentamers, in contradiction to grape seed that shows major oligomers corresponding to molecules with increased DPm [16]. High DPm molecules are most likely to be the active principles of these compounds [25]. Likewise, reduction of the interfibrillar space caused by short-length molecules could lead to decreased water sorption [15] and therefore poor infiltration of resin monomers. Another important factor would be the PA content of each extract. The lowest level of PA presented in CSE (about 45%) when compared with the GSE (at least 95%) could slow down or even decrease their ability to interact with collagen. The last potential interference in extracts effectiveness could be inherent to the solvents. Acetone and ethanol from CSE groups could cause dehydration of dentin matrix, stabilizing the collagen structure by temporarily increasing the amount of interactions of weak forces between adjacent collagen molecules hampering, even more, the penetration of the adhesive [26]. 

Some kind of relationship between PA agents and adhesives systems could be inferred. GSE showed a more predictable behavior when SB, an ethanol-water adhesive, was applied and CSE demonstrated enhanced results with OS, an acetone based adhesive. This is probably due to a greater affinity among solvents from adhesives and solutions; both ethanol and acetone are polar solvents miscible in water. However, ethanol is a protic solvent solvating anions (negatively charged solutes) strongly via hydrogen bonding just like water. Meanwhile acetone is an aprotic solvent with large dipole moments (separation of partial positive and partial negative charges within the same molecule) and solvates positively charged species via their negative dipole [27], with decreased Hansen's hydrogen bond (*δ*H) parameter (7.0) which is lower than water *δ*H (42.3) and ethanol (19.4) [28]. So, it is reasonable to predict that solvent system used in each solution interacts differently with diverse solvents from the adhesive system. 

The type of adhesive also affected results after 12 months artificial saliva storage. Hashimoto [29] showed in a recent study that the degradation after 1 year storage occurs for all members of the HL and whilst SB is extremely hydrophilic; nanoleakage concentration of silver nitrate grains was found only in the adhesive layer, maintaining the HL intact. OS specimens showed resin missing from the interfibrillar space of the collagen, resulting in the creation of microvoids within the HL under SEM [29]. This could reduce long-term *μ*TBS values when OS was the system of choice, what could be verified by the highest percentage of *μ*TBS degradation (Figure 1).

Interestingly, no statistical difference was observed in the percentage of *μ*TBS reduction regarding dentin treatment, although CSE/ET and CSE/AC presented the lowest variation for both adhesives, and it is probably because ethanol and acetone decrease even more the dielectric constant of the media, stimulating PA and collagen interactions for long periods [11]. Another revealing result is the highest percentage reduction of GSE's *μ*TBS; however, due to its great immediate results, long-term results were satisfactory. Their potential interaction with noncollagenous proteins such as proteoglycans and matrix metalloproteinases [30] may affect the characteristics of the tissue. Proanthocyanidin, for example, is a potential inhibitor of MMP-2 and MMP-9 [31]. Current literature suggests that this MMP resistance may be attributed to an alternative mechanism-silencing of MMPs and probably other exogenous collagen degradation enzymes via conformational changes in the enzyme 3D structure [32]. The use of cross-linking agents may also contribute to MMP silencing via allosteric control of noncatalytic domains [33]. 

GSE has unique ability to biomodify dentin matrix most likely by presence of exogenous cross-links which could probably develop a stronger and more durable substrate, without reducing the resin penetration and consequently improving the short- and long-term *μ*TBS. CSE presented more stable values over one year analysis. However, one year storage period in artificial saliva may not be enough challenge for current adhesive systems, and maybe increasing storage time and/or adding collagenolytic enzymes could provide greater challenges and better reflect clinical need for these systems. Biodegradation analyses of the interface such as nanoleakage and more complex analysis of extracts in order to find the active principles and determine their role in the properties and stability of dentin collagen are ongoing. 

## 8. Conclusion 

Collagen is a component of HL; therefore, the stabilization and improvement of mechanical properties could generate a more suitable substrate for adhesive restorations. Bio-modification of dentin matrix, by induction of exogenous cross-linking, using PA-based extracts may contribute to increased micromechanical interlocking and long-term resin-dentin bonds. 

## Figures and Tables

**Table 1:** Composition and batch numbers of used materials.

Material (manufacturer)	Composition or PA content	Batch number
Filtek Supreme Plus (3M/ESPE, St. Paul, MN, USA)	Bis-GMA, UDMA, TEGDMA, bis-EMA, zirconia, and silica filler	20070625

Adper Single Bond Plus (3M/ESPE, St. Paul, MN, USA)	Bis-GMA, HEMA, dimethacrylates, polyalkenoic acid copolymer, initiators, silica nanofiller, water, and ethanol	6HL

One Step Plus (Bisco, Inc., Schaumburg, IL, USA)	Bis-GMA, HEMA, BPDM, initiator, dental glass, and acetone	0700010854

Grape seed extract (Mega-Natural Gold grape seed extract, Polyphenolics Madera, CA, USA)	95% PA content*	13682503-01

Cocoa seed extract (polyphenol, Foratero, Barry Callebaut, Lebbeke-Wieze, Belgium)	45% PA content*	CMIE-7LJJKF

*Information provided by the manufacturer.

PA: proanthocyanidins.

**Table 2:** Bond strength (MPa) of demineralized dentin treated (standard deviation) with different dentin biomodification agents in different application times and storage periods for Single Bond Plus (SB) and One Step Plus (OS). One-way ANOVA *P* values for different statistical groups.

Adhesive system	Variables	*μ*BS (MPa)				*P*
		Storage time				
		24 hours	3 months	6 months	12 months	
SB	GSE					
	10 min	65.96 (12.36)^Aa^	64.17 (16.72)^Aa^	64.17 (16.16)^Aa^	57.15 (15.29)^Aa^	0.185
	CSE/ET					
	10 min	56.95 (14.40)^Aab^	55.90 (14.11)^Aab^	52.88 (11.46)^Ab^	54.04 (17.12)^Aa^	0.753
	CSE/AC					
	10 min	49.97 (11.91)^Ab^	51.75 (16.82)^Ab^	49.98 (13.97)^Ab^	48.22 (19.46)^Aa^	0.890
	Control					
	10 min	54.51 (20.03)^Ab^	52.74 (16.31)^Aab^	49.16 (13.88)^Ab^	51.68 (19.42)^Aa^	0.759
	*P*	0.003	0.031	0.001	0.347	

OS	GSE					
	10 min	60.93 (8.79)^Aa^	53.57 (12.4)^Ba^	50.41 (10.14)^Ba^	42.62 (7.46)^Cab^	0.000
	CSE/ET					
	10 min	60.28 (12.86)^Aa^	55.18 (11.26)^Aa^	47.01 (8.25)^Bab^	44.6 (10.95)^Ba^	0.000
	CSE/AC					
	10 min	54.44 (11.01)^Aab^	55.76 (9.70)^Aa^	48.90 (16.50)^ABab^	41.30 (6.83)^Bab^	0.000
	Control					
	10 min	50.39 (18.17)^Ab^	49.66 (9.38)^Aa^	39.11 (12.71)^Bb^	36.85 (10.41)^Bb^	0.000
	*P*	0.016	0.187	0.011	0.036	

GSE: grape seed extract; CSE: cocoa seed extract; ET: ethanol; AC: acetone; *P*: probability value. Different lower and upper case letters show statistical differences among rows and columns, respectively. Note: adhesive systems were not compared with each other.

**Table 3:** Two-way ANOVA for both adhesive systems.

Adhesive	Source	DF	MSR	P
SB	Storage	3	1.38	0.250
	Treatment	3	12.73	0.000
	Storage ∗ Treatment	9	0.38	0.947

OS	Storage	3	35.31	0.000
	Treatment	3	10.04	0.000
	Storage ∗ Treatment	9	0.86	0.563

One Step Plus (OS) and Singe Bond Plus (SB).

DF: degree of freedom; MSR: mean square value; *P*: probability value.
